# Escaping the Jingle-Jangle Jungle: Increasing Conceptual Clarity in Psychology Using Large Language Models

**DOI:** 10.1177/09637214251382083

**Published:** 2025-10-22

**Authors:** Dirk U. Wulff, Rui Mata

**Affiliations:** 1 Max Planck Institute for Human Development, Berlin, Germany; 2 Center for Cognitive and Decision Sciences, Faculty of Psychology, University of Basel

**Keywords:** taxonomic incommensurability, conceptual clarity, embeddings, large language models

## Abstract

Psychology has long struggled with conceptual redundancy, particularly in the form of “jingle-jangle fallacies,” in which different constructs share the same label or the same construct is described using different terms. This lack of conceptual clarity has hindered cumulative knowledge and comparability across studies and subfields. We propose that large language models can help address this issue by placing constructs into a shared semantic space, enabling the systematic mapping of conceptual overlap and clarification of taxonomies and generating clearer construct definitions. Although automation plays a crucial role, we argue that meaningful progress requires a coordinated, community-wide effort, combining computational advances with expert deliberation. Our approach provides a pathway toward greater conceptual clarity in psychology, fostering a more unified and rigorous framework for the discipline.

Today, most of us take the periodic table of elements for granted, yet its creation was not the result of linear, cumulative progress. It evolved through successive refinements, with elements systematically introduced, reclassified, or eliminated as scientific understanding of physical matter advanced. For example, early chemical theories proposed the element “phlogiston,” believed to be a substance released during burning, to account for combustion phenomena but were ultimately discarded in light of the oxygen theory. Similarly, the proposed element “newtonium,” thought to penetrate all substances (an homage to Newton’s widespread scientific influence), was discarded when the scant supporting evidence was shown to stem from methodological errors. To date, more proposed elements have been discarded than the 118 elements that make up the periodic table (Fontani et al., 2014). Chemistry thus illustrates a path from hypothesizing elements to rigorously testing and, crucially, disproving their existence.

Psychology, like chemistry, is no stranger to the notion of “lost elements.” Diagnoses such as hysteria and “drapetomania” were eventually abandoned after being exposed as being rooted in sexism and racism rather than scientific evidence (e.g., Bynum, 2000; Tasca et al., 2012). Yet unlike chemistry, which developed systematic methods for refining and eliminating invalid elements, psychology continues to see unchecked growth (e.g., Anvari et al., 2025; Bringmann et al., 2022; Elson et al., 2023; Leising et al., 2022; Sharp et al., 2023; Wulff & Mata, 2025). This conceptual proliferation creates practical problems for a wide range of stakeholders: Researchers struggle to select appropriate measures given largely overlapping constructs, consortia face difficulties integrating theory and data because of inconsistent definitions, editors and funders lack clear standards for assessing the novelty of proposed constructs, and professional organizations struggle to build coherent training frameworks and unified taxonomies (i.e., systematic classification frameworks that organize constructs and provide shared standards for definition and measurement). Without the conceptual pruning that supports cumulative progress, psychology risks being a discipline crowded with redundant or ill-defined elements.

In this article, we discuss how psychology can profit from recent methodological advances to address these issues. Specifically, we argue that large language models can help provide a systematic and scalable approach for detecting and resolving conceptual redundancies that have plagued the field. We first outline the problem of jingle-jangle fallacies in psychology. We then introduce a general computational approach and discuss technical considerations, practical issues, and limitations. Last, we discuss the broader research landscape necessary for implementing these solutions effectively and equitably.

## Welcome to the Jingle-Jangle Jungle

**Figure 1.**
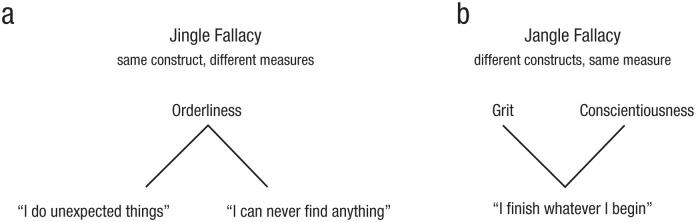
Distinction between jingle and jangle fallacies based on existing constructs and measures in personality research. The (a) jingle fallacy refers to the use of the same construct label, “orderliness,” for different measures (typically a collection of items), here exemplified by the statements “I do unexpected things” and “I can never find anything.” Although both measures are labeled “orderliness,” they may capture distinct aspects of behavior, such as spontaneity versus orderliness per se. The (b) jangle fallacy occurs when different construct labels, “grit” and “conscientiousness,” are used for the same underlying measure (typically a collection of items), here exemplified by the statement “I finish whatever I begin.”

The problem of lack of conceptual clarity in the form of a clear mapping between constructs and operationalizations or measures has long been acknowledged in psychology and discussed under the banner of so-called jingle-jangle fallacies, that is, two types of conceptual confusion that arise in psychological research. The jingle fallacy occurs when two (or more) measures are mistakenly labeled with the same construct label, leading researchers to assume that they are equivalent when, in fact, they capture distinct phenomena (Aikins, 1902; see Fig. 1a). Conversely, the jangle fallacy involves using different construct labels to describe the same measure, giving the false impression that these construct labels represent unique constructs (Kelley, 1927; see Fig. 1b).

Unfortunately, recent evidence suggests that conceptual ambiguity is worsening in psychology, with a growing proliferation of both constructs and measures over the past decades. Elson et al. (2023) analyzed the American Psychological Association’s PsycTests database and found more than 38,000 constructs and 43,000 measures, most of which are used only infrequently. This pattern contributes to fragmentation rather than standardization and highlights substantial potential for conceptual and measurement overlap in the field.

Ambiguities in how psychological constructs map onto their measures can arise when different research traditions adopt different methods and connect theories to observations in incompatible ways—a problem philosophers call “taxonomic incommensurability” (Oberheim & Hoyningen-Huene, 2018). This fragmentation is compounded by a “division of linguistic labor,” as key terms take on divergent meanings across subfields, impeding cumulative progress (Putnam, 1973). Although such issues are widely recognized, psychology has lacked scalable tools to quantify and manage conceptual engineering—the systematic refinement of conceptual frameworks (Chalmers, 2020). Past approaches involved extensive construct validation, for example, by conducting prediction studies to assess the differential predictive validity of various constructs (Cronbach & Meehl, 1955) or by triangulating across multiple constructs using costly multitrait, multimethod designs to establish convergent and discriminant validity (Campbell & Fiske, 1959). However, because such studies are resource intensive, they have been rare and typically focus on a narrow subset of constructs and measures, leaving considerable gaps unresolved. Today, large language models can help automate parts of this process by mapping constructs into shared semantic spaces that empirically map relationships between all extant constructs in a relatively easy way, enabling the detection and resolution of conceptual redundancies at scale.

## Increasing Conceptual Clarity With Large Language Models

Large language models provide us with semantic embeddings—high-dimensional representations of words or phrases that capture meaning on the basis of contextual relationships—offering a promising approach to clarifying the connections between psychological constructs. Earlier studies have demonstrated the utility of semantic embeddings for mapping and differentiating constructs in psychology (e.g., Larsen & Bong, 2016; Rosenbusch et al., 2020). However, recent advances have dramatically enhanced the capacity of these models, making them powerful tools for addressing taxonomic incommensurability (e.g., Hussain et al., 2024; Wulff & Mata, 2025).

Recently, Wulff and Mata (2025) leveraged semantic embeddings of item, scale, and labels from a large set of personality measures to model the semantic relationships between thousands of psychometric items and hundreds of scales and labels. Their analysis demonstrated that semantic embeddings could effectively predict empirical associations between items and scales while also automatically detecting jingle-jangle fallacies in construct assignments. Additionally, they introduced methods to refine psychological taxonomies by systematically reducing redundancy through reassigning labels to scales. In one approach, they proposed a streamlined version of personality measures with 75% fewer hypothesized constructs, highlighting the potential of semantic embeddings to create more parsimonious and coherent taxonomies in psychology.

Although the work discussed above focuses on personality psychology, we argue that this approach is broadly applicable across the discipline. Advancing the field as a whole will require extending such methods to other psychological domains, in which similar issues of conceptual ambiguity and redundancy persist. We outline three main classes of applications in which large language models can be used to promote conceptual clarity.

**Figure 2.**
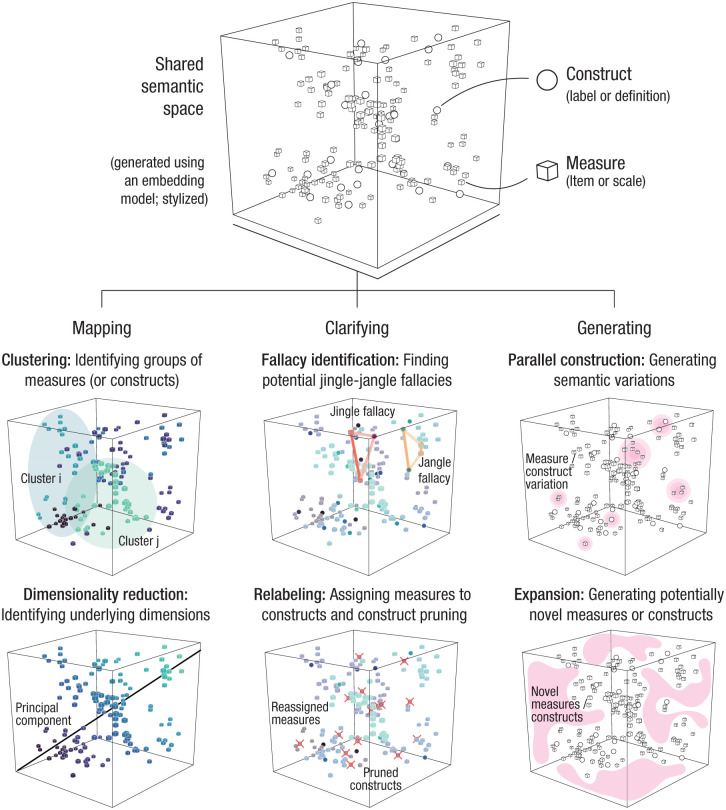
General computational approach to enhancing conceptual clarity in psychology. Constructs (e.g., labels or definitions) and measures (e.g., items or scales) from the psychological literature are embedded into a shared semantic space (top cube). In this example, we use stylized data generated from an embedding model and apply different analytical techniques. This semantic space serves as the foundation for three classes of applications illustrated in the lower part of the figure: mapping, clarifying, and generating. **Mapping:** *Clustering* groups measures to uncover the latent structure and identify redundancy (colors indicate the cluster to which each measure belongs); *dimensionality reduction* highlights the key dimensions of variation (the color of each measure indicates its factor loading on a principal component). **Clarifying:** *Fallacy detection* identifies conceptual overlap through the detection of jingle fallacies (colored lines link the same construct label to two different measures) and jangle fallacies (colored lines connect two distinct construct labels to two similar measures); *relabeling* reassigns or relabels measures and prunes redundant constructs to refine the taxonomy structure (crosses represent pruned constructs). **Generating:** *Parallel construction* uses a generative model to produce semantically aligned variations of existing measures; *expansion* represents the use of a generative model to create new items or constructs that address conceptual gaps.

Figure 2 illustrates an approach in which psychological measures and constructs are placed into a common semantic space, allowing researchers to apply many of the same analytical strategies long familiar in psychology—such as clustering or dimensionality reduction—to semantic embeddings rather than purely to empirical associations. One important class of applications concerns *mapping* the relations between existing measures and constructs. For example, clustering allows researchers to group semantically similar measures and constructs to detect redundancies, identify overlapping concepts, and uncover latent relationships between different measures. Thus, researchers can systematically examine the relations between measures and constructs using strategies envisioned in earlier work on construct validation (e.g., Cronbach & Meehl, 1955), but now at a greater scale and with greater efficiency. This provides a new basis for making more precise and transparent decisions about measurement strategies, improving conceptual clarity in psychology.

A second class of applications involves *clarifying* existing taxonomies, for example, by using semantic embeddings to systematically reassign construct labels on the basis of their semantic similarity to established or novel concepts. This method may be particularly useful for standardizing terminology across studies or even subfields, ensuring greater consistency and reproducibility in psychological research. By aligning construct labels with empirical and theoretical relationships, researchers can reduce ambiguity, resolve jingle-jangle fallacies, and promote clearer communication within the field. Crucially, instead of relying on arbitrary naming conventions, this approach offers a replicable, data-driven method for refining psychological taxonomies.

Last, a third class of applications involves *generating* new measures or constructs. Although this may appear counterintuitive to the goal of reducing conceptual proliferation, generative language models can support clarity in at least two ways. First, they can assist in creating psychometric items that are semantically well aligned with existing constructs, enhancing internal consistency and content coverage without resorting to loosely related or conceptually distant alternatives. Second, they can help identify gaps in the conceptual landscape, guiding the development of constructs that address blind spots rather than duplicating existing ideas. Crucially, because these generative outputs can be situated within the same semantic space used for mapping and clarification, they can be evaluated with comparable rigor—ensuring that new contributions enhance, rather than dilute, conceptual clarity.

## Technical Considerations, Practical Issues, and Limitations

Although the mapping, clarifying, and generating applications illustrated in Figure 2 share a common foundation, their implementation raises technical considerations. First, different applications require distinct technical uses of large language models. Most of the applications discussed above concerning mapping and clarification tasks involve obtaining embeddings from large language models (i.e., feature extraction) and using these embeddings to locate constructs in high-dimensional semantic spaces (e.g., Abdurahman et al., 2024; Hommel & Arslan, 2024; Wulff & Mata, 2025). One technical question with this approach is the type of embeddings to be used: General-purpose embeddings capture broad semantic knowledge, whereas embeddings obtained from training models to specific tasks (fine-tuning) offer task-specific sensitivity but may risk reduced generalizability. Other applications require different models or setups that involve generating new items or constructs through prompting (i.e., text generation; Götz et al., 2024). The different models and approaches may not always provide equivalent results; thus, future work will need to assess which are most suitable for different applications (Achille et al., 2019; Demircan et al., 2024). Integrating these different uses will be crucial to creating productive pipelines that integrate both generation and evaluation to avoid unwarranted proliferation of measures or constructs (Laverghetta et al., 2024).

There are important practical issues, particularly given the complex evolving landscape of models, approaches, and stakeholders. Table 1 outlines examples of potential uses—some of which are already accessible through tutorials (Wulff et al., 2024) or online applications (Hommel & Arslan, 2024; Rosenbusch et al., 2020)—by different stakeholders. Improving this potential, however, depends on three conditions. First, adequate infrastructure is required. Although large language models are widely available as chatbots, the type of academic applications discussed demand access to resources that are not currently available to all, particularly those involving open-weight models (the internal parameters of which are publicly available) that require dedicated infrastructure to support data sovereignty, transparency, and reproducibility (Wulff et al., 2024). Second, researchers need appropriate training. Although some uses can be implemented via online tools, more advanced uses require additional efforts, including hands-on workshops and integration into academic curricula. Third, professional organizations must play a role in supporting adoption, for example, by creating dedicated task forces to deliberate (and communicate) on best practices for integrating these tools into both theoretical development and applied work.

**Table 1.** Example Strategies by Stakeholder

Stakeholder	Mapping	Clarifying	Generating
Researchers	Cluster measures to detect semantic overlap or use dimensionality reduction to inform measure selection for primary studies.	Detect and remove jingle-jangle fallacies in construct measure assignments when planning a study or interpreting results.	Use generative models to create new items for studies involving multiple measurement occasions.
Consortia	Compare taxonomies across research areas to identify conceptual overlaps or silos to improve collaboration efforts.	Harmonize terminology across subfields by realigning measures and constructs.	Develop domain-specific measurement frameworks to fill gaps in emerging or interdisciplinary areas.
Editors and funders	Evaluate semantic similarity of new constructs or measures to existing literature to assess novelty.	Flag ambiguous or redundant measures during review using automated semantic checks and request clarification from authors.	Encourage development of measures or constructs in special issues or calls in semantically underrepresented areas to promote innovation and conceptual balance.
Professional organizations	Use embeddings to continuously track and visualize construct proliferation and clustering trends in the field.	Standardize definitions and update taxonomies by consolidating semantically similar entries in psychological dictionaries.	Support tools for generating candidate constructs or organizing frameworks to guide future research.

There are a number of limitations. First, semantically similar constructs should not always be treated as necessarily identical. For example, psychology has a tradition of distinguishing between subjective experience and objective states (e.g., subjective stress vs. cortisol stress response) that may be semantically related but potentially theoretically distinct. The extent to which such cases abound and are (or are not) captured by large language models remains to be assessed. Second, relatedly, most work has focused on text-based measures, such as personality items and self-reports, which align well with language-based models (e.g., Hommel & Arslan, 2024; Wulff & Mata, 2025) but represent only a small portion of measurement in psychology. Recent successes in predicting behavioral outcomes using large language models suggest potential for extending these methods to task-based measures (Binz et al., 2025), but current limitations in cross-measure integration must be overcome to support a comprehensive map of constructs and promote conceptual clarity across psychology.

## Moving Forward: Large Language Models Should Inform, Not Replace, Expert Debate

Large language models do not offer a value-neutral vision of the conceptual landscape of psychology because their outputs inevitably reflect the biases of their training data and regimes; thus, one cannot propose using large language models to enforce conceptual uniformity from a neutral perspective. Furthermore, construct diversity can reflect genuine differences in theoretical orientation or measurement aims that serve divergent but legitimate purposes across research communities. Consequently, efforts to consolidate constructs must recognize the epistemic value of pluralism (e.g., Hochstein, 2016; Iliescu et al., 2024) and keep in mind that decisions about constructs that are retained, merged, or eliminated are rarely neutral but shaped by the priorities of specific interest groups and sociohistorical norms (e.g., Danziger, 2013). These considerations suggest that the use of automated methods can at best be thought of as facilitating the systematic evaluation of the construct landscape and supporting transparent negotiation among stakeholders through structured dialogue. We believe this aligns well with past calls for greater conceptual clarity, which have emphasized the role of expert coordination and shared infrastructure to address construct proliferation (e.g., Anvari et al., 2025; Bringmann et al., 2022; Sharp et al., 2023).

When discussing the idea of conceptual engineering, Chalmers (2020) argued that aspects of concept design and evaluation, such as those we introduced above regarding the overlap of different constructs, are relatively tractable. However, widespread concept implementation—that is, actual change in the use of concepts (e.g., consistent adoption of a specific measure to capture a construct)—is a more difficult social project. The field of psychology has seen recent developments that leave us optimistic, including the growing adoption of reporting standards, preregistration, and related reforms. Of course, any proposal that gives researchers, editors, and professional organizations greater influence over the use of terms and measures may conflict with ideals of diversity and freedom. We hope that our proposal, when embedded within a broader framework of collaborative and critically reflexive debate, can help balance the goals of coherence and diversity in psychology, with large language models serving as tools to support—not dictate—this process.

## Conclusion

Large language models offer scalable tools for detecting, clarifying, and generating psychological constructs within a shared semantic space. As a form of conceptual engineering, this approach can inform expert deliberation and support collaborative refinement of conceptual landscapes. Although not a substitute for shared deliberation, these tools provide a principled means for advancing conceptual clarity in psychology.

## Recommended Reading

Bringmann, L. F., Elmer, T., & Eronen, M. I. (2022). (See References). Makes a conceptual argument for the importance of conceptual clarification in psychology.

Elson, M., Hussey, I., Alsalti, T., & Arslan, R. C. (2023). (See References). Provides an empirical overview of the proliferation of constructs and measures in psychology.

Hussain, Z., Binz, M., Mata, R., & Wulff, D. U. (2024). (See References). Introduces large language models as tools for psychological research, including how these models can be used for mapping, clarifying, and generating goals in psychology.

Wulff, D. U., & Mata, R. (2025). (See References). Offers a solution to identifying and resolving jingle-jangle fallacies on the basis of semantic embeddings obtained from large language models.

## Transparency

*Action Editor:* Wiebke Bleidorn

*Editor:* June Gruber
